# Whole chromosome loss and genomic instability in mouse embryos after CRISPR-Cas9 genome editing

**DOI:** 10.1038/s41467-021-26097-y

**Published:** 2021-10-06

**Authors:** Stamatis Papathanasiou, Styliani Markoulaki, Logan J. Blaine, Mitchell L. Leibowitz, Cheng-Zhong Zhang, Rudolf Jaenisch, David Pellman

**Affiliations:** 1 Department of Cell Biology, Blavatnik Institute, Harvard Medical School, Boston, MA USA; 2 Department of Pediatric Oncology, Dana-Farber Cancer Institute, Boston, MA USA; 3 Whitehead Institute, Cambridge, MA USA; 4 Howard Hughes Medical Institute, Chevy Chase, MD USA; 5 Department of Biomedical Informatics, Harvard Medical School, Boston, MA USA; 6 Department of Data Sciences, Dana-Farber Cancer Institute, Boston, MA USA; 7 Massachusetts Institute of Technology, Department of Biology, Cambridge, MA USA

**Keywords:** Chromosome segregation, CRISPR-Cas9 genome editing, Genomic instability

## Abstract

Karyotype alterations have emerged as on-target complications from CRISPR-Cas9 genome editing. However, the events that lead to these karyotypic changes in embryos after Cas9-treatment remain unknown. Here, using imaging and single-cell genome sequencing of 8-cell stage embryos, we track both spontaneous and Cas9-induced karyotype aberrations through the first three divisions of embryonic development. We observe the generation of abnormal structures of the nucleus that arise as a consequence of errors in mitosis, including micronuclei and chromosome bridges, and determine their contribution to common karyotype aberrations including whole chromosome loss that has been recently reported after editing in embryos. Together, these data demonstrate that Cas9-mediated germline genome editing can lead to unwanted on-target side effects, including major chromosome structural alterations that can be propagated over several divisions of embryonic development.

## Abstract (web summary)

A possible undesired outcome of CRISPR-Cas9 germline editing is unwanted karyotype alterations. Here the authors track aberrations through three divisions of embryonic development following Cas9 editing.

## Introduction

The implementation of CRISPR-Cas9 for germline genome editing holds promise for therapy for genetic diseases^1,2^. However, the risks associated with germline editing are only just starting to be understood^1^. In addition to mosaicism and off-target mutations^1^, recent work has identified on-target adverse outcomes in embryos. Specifically, Adikusuma et al.^3^ observed deletions of up to 2.3 kb near the target site in mouse embryos. Furthermore, after editing of human pre-implantation embryos, several studies identified larger, megabase-scale deletions^4,5^. Counterintuitively, CRISPR-Cas9 editing in embryos also commonly leads to the loss of the entire targeted chromosome^5^, the cause of which is unclear.

We recently showed that Cas9-induced double strand breaks (DSB) can generate micronuclei and chromosome bridges in human cell lines and primary blood stem and progenitor cells^6^. These structures can trigger catastrophic mutational processes called chromothripsis and the chromosome breakage-fusion-bridge cycle^6–11^. Here, we show that CRISPR-Cas9 DSBs lead to similar mitotic errors in the mouse pre-implantation embryo and elucidate how CRISPR-Cas9 can lead to whole chromosome loss.

## Results

### Cas9 induces micronuclei and aneuploidy in mouse embryos

**Fig. 1:**
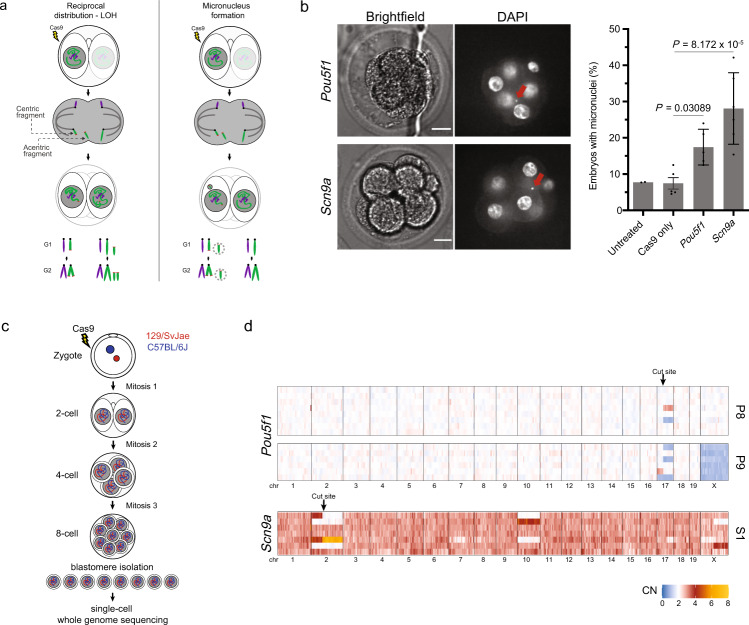
Copy number alterations and micronucleation from CRISPR-Cas9 genome editing in mouse embryos. **a** Schematic of two chromosome segregation errors following Cas9 treatment in cleavage stage mouse embryos. Left, missegregation of the Cas9-induced acentric fragment to one daughter and segregation of the centric fragment to the other generates a reciprocal gain/loss copy number (CN) pattern. LOH is loss of heterozygosity. Right, the acentric fragment segregates to the same daughter as the centric fragment but is partitioned into a micronucleus. Because of defective DNA replication in the micronucleus, in G2 cells, the acentric fragment will have ~0.5 CN of the centric fragment. Targeted chromosome: green; normal chromosome: purple. Cas9 cut site is marked in red. **b** Micronucleus formation after CRISPR-Cas9 treatment. Left, representative single z-focal plane confocal images of 8-cell stage mouse embryos after CRISPR-Cas9 with *Pou5f1* and *Scn9a* gRNAs that are quantified in the right panel. Red arrows: micronuclei. Right, percentage of 8-cell stage embryos with at least one micronucleus (*n* = 2, 5, 5, 6 experiments with 77, 97, 136, and 99 embryos, left to right). Error bars: mean ± SEM, two-tailed Fisher’s exact test against the “Cas9 only” group. Scale bars: 15 μm. Source data are provided as a Source Data file. **c** Schematic of the experimental strategy for single-cell whole-genome DNA sequencing. **d** Heatmap representing total CN for each chromosome for the P8 and P9 embryos after CRISPR-Cas9 treatment with the *Pou5f1* and S1 with the *Scn9a* gRNAs. Bin size: 5 Mb. Arrows indicate the genomic location of the targeted cleavage site for the indicated gRNAs. Note: the S1 embryo is triploid (see main text and Supplementary Data 1).

A Cas9-mediated DSB divides a chromosome into two fragments, one of which contains a centromere (the centric fragment) and another that does not (the acentric fragment). If unrepaired by the time of mitotic entry, the acentric fragment is prone to chromosome missegregation and micronucleus formation^12^ (Fig. 1a). To assess micronucleation after CRISPR-Cas9 editing in embryos, we subjected mouse zygotes to single-guide CRISPR-Cas9 ribonucleoprotein electroporation using two different gRNAs targeting unique loci on chromosome 2 and chromosome 17 (see Methods section). A gRNA targeting the second exon of *Pou5f1* was selected because it has been extensively characterized in mouse embryo editing experiments^2,13^. The gRNA targeting an intronic sequence of *Scn9a* in chromosome 2 was selected because of the large distance between the cut site and the telomere, which should generate a large, easily detectable acentric fragment (116 Mb). Indeed, micronucleus formation in 8-cell stage embryos was increased relative to controls without gRNAs (2.3-fold for the *Pou5f1* and 3.8-fold for the *Scn9a* guides, Fig. 1b). As expected from prior reports^14^, we observed a 7.7% baseline frequency of spontaneously formed micronuclei in 8-cell stage mouse embryos.

We next evaluated chromosomal alterations genome-wide following single-guide Cas9/gRNA ribonucleoprotein delivery into the zygote. After editing, we isolated the individual blastomeres from 8-cell stage embryos and performed single-cell whole-genome sequencing (Fig. 1c). Because haplotype information markedly enhances copy number (CN) analysis in single-cell genomes, we used zygotes from a hybrid cross (129/SvJae x C57BL/6J). In total, we performed low-pass whole genome sequencing (average of 0.15X genome coverage) on 202 cells derived from 24 embryos electroporated with the same gRNA/Cas9 RNPs listed above. Three untreated embryos were used as controls (Supplementary Fig. 1a and Data 1). We then calculated genome-wide CN estimates in 5 Mb bins for all the blastomeres for which sufficiently high-quality libraries were generated (174/202 libraries, see Methods section).

We identified three embryos (12.5%) with arm-level CN alterations that initiate at the gRNA target site: two embryos from the *Pou5f1* gRNA group (P8 and P9, 17% of the total *Pou5f1* gRNA-targeted embryos) and one from the *Scn9a* group (S1, 8% of *Scn9a* gRNA-targeted embryos) (Fig. 1d). In both the P8 and P9 embryos the only copy number alterations that were detected were associated with the Cas9 cleavage of the targeted locus.

**Fig. 2:**
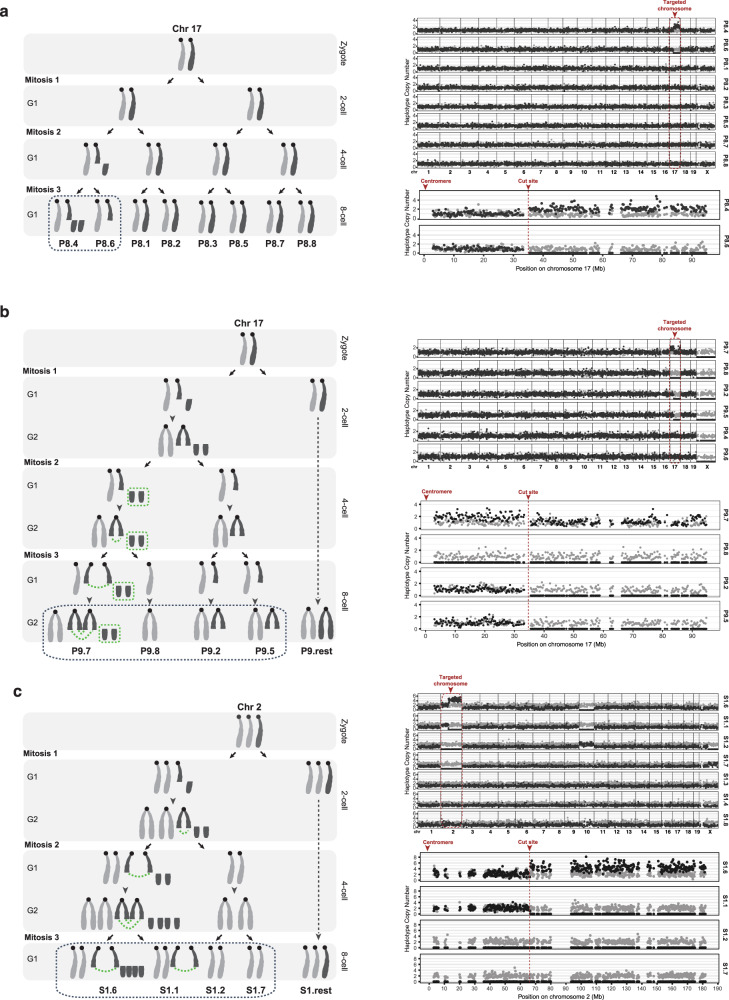
Complex karyotype alterations and whole chromosome monosomy after CRISPR-Cas9 treatment in embryos. **a** Left, illustration of the inferred karyotype alterations during the development of the P8 embryo (*Pou5f1* gRNA). Chromosomes are shaded to indicate parental haplotype. Right top: relative haplotype copy number scatter plots for all chromosomes in all blastomeres (1 Mb bins, dark gray dots: 129/SvJae; light gray dots: C57BL/6J). Black dotted boxes: blastomeres shown in the bottom right panel; Right bottom: zoomed view of relative haplotype copy number plots for the targeted chromosome (250 kb bins). **b** P9 embryo (*Pou5f1* gRNA), as in (**a**) above. Green dotted box: micronucleus; green dotted curves: chromosome bridges. The acentric chromosome fragment of the targeted chromosome 17 forms a micronucleus at the 4-cell stage and undergoes poor DNA replication. Fusion of the break ends of the centric fragments generates a dicentric chromosome. CN data suggest that this embryo, unlike the others, was harvested with its cells in the G2 phase, which is why the diagram on the left illustrates CN values of either two or four. The CN plots on the right have been normalized to one, and therefore have values of either one or two. See Supplementary Fig. 4 for alternative explanations of some features of the CN patterns. **c** S1 embryo (*Scn9a* gRNA), as in (**a**) and (**b**) above. The centric fragments of the targeted chromosome form a chromosome bridge at the 2-cell stage and all the acentric fragments missegregate in mitosis 2 and 3 to the daughters containing the centric fragments. Notes: the Cas9 cut in (**b**) and (**c**) can be introduced either on a single chromatid in G1 or in both sister chromatids in G2 (shown here is G1 cleavage). See Supplementary Fig. 4 for alternative explanations of some features of the CN patterns.

In total, we identified 10 blastomeres (from P8, P9, and S1 embryos) that experienced missegregation of the acentric fragment or the entire chromosome containing the CRISPR-Cas9 target site (Fig. 2). Whole chromosome loss (monosomy) was observed in three blastomeres (P9.8, S1.2, and S1.7). Unexplained loss of the entire targeted chromosome has been previously reported after Cas9 genome editing in human embryos^5^.

In addition to the CN alterations of the targeted chromosome, we also observed missegregation events affecting other chromosomes (Supplementary Fig. 1 and Data 1). This is expected from the previously reported baseline rate of cell division errors during early development^5,15,16^. In four embryos the CN alterations affected an entire chromosome, whereas 10 embryos exhibited arm-level CN alterations. The breakpoints for the arm-level CN alterations did not occur at predicted CRISPR-Cas9 off-target sites (Supplementary Fig. 2).

### Reconstructing karyotypic history of 8-cell stage embryos

We performed deeper sequencing (average of 9.45X coverage) of 13 embryos (99 blastomeres, both gRNA groups) that displayed arm-level CN alterations in at least one blastomere (“NovaSeq” samples, Supplementary Data 1). CN alterations shared between blastomeres of a given embryo involved the same haplotype, consistent with the generation of acentric chromosome fragments followed by their asymmetric distribution between progeny cells. Other events could also be deconvolved using the haplotype CN analysis. For example, chromosome 5 and X trisomies, shared in all S7 embryo blastomeres, presumably reflect missegregation events during maternal meiosis (Supplementary Fig. 1). Additionally, we observed two digynic embryos (S1 and P2, Supplementary Fig. 1) also resulting from maternal meiotic cell division errors, a common source of embryonic aneuploidy and mosaicism^17^.

**Fig. 3:**
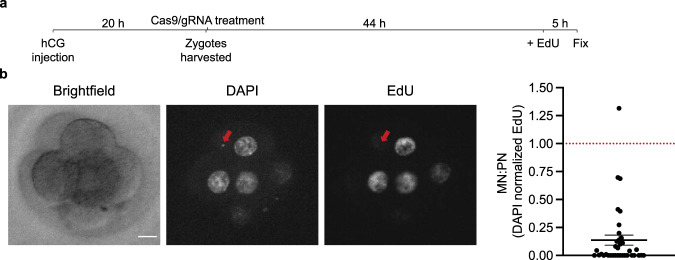
Micronuclei in embryonic cells undergo defective DNA replication. **a** Scheme for the experiment. hCG human Chorionic Gonadotropin. **b** Left, representative single z-focal plane image of blastomeres, quantified in the right panel, by brightfield confocal microscopy and corresponding fluorescence DAPI and EdU-labeling. The red arrow points to a micronucleus (MN) detected by DAPI staining that fails to incorporate EdU, despite active replication in the primary nucleus (PN). Scale-bar: 10 μm. Right, quantification of the ratio of density of EdU signal in micronuclei relative to primary nuclei. If micronuclei replicate normally this ratio will be one (dotted red line). Strongly reduced DNA replication was evident in most micronuclei. Error bars: mean ± SEM (*n* = 1 experiment with 36 micronuclei scored). Source data are provided as a Source Data file.

Chromosomes in micronuclei undergo poor DNA replication which is readily detected by single-cell DNA sequencing, as we observed in our prior work in cell lines^6,7,10^. Consistent with the micronuclear DNA replication defect being a general phenomenon, by incorporation of 5-ethynyl-2′-deoxyuridine (EdU) in newly synthesized DNA of live embryos, we showed that micronuclei generated in embryos after CRISPR-Cas9 treatment also undergo significantly defective DNA replication, (*p* < 0.0001, two-sided Mann–Whitney test of micronucleus “MN” compared to primary nucleus “control” group, Fig. 3). From sequencing, we identified an example (P9 embryo) where an acentric fragment from the targeted chromosome was underreplicated and therefore originated from a micronucleus (Fig. 2b). By this criterion, micronuclei formed in two other embryos, but in these cases were derived from non-targeted chromosomes (whole chromosome 10 in S1 and chromosome 3 fragment in S8, Supplementary Fig. 3). These micronuclei likely derive from spontaneous missegregation events that commonly occur during early development^14^ (Fig. 1b). It is also possible, as has recently been proposed, that Cas9 on its own, without a gRNA, might create some DSBs^18^.

We also identified CN alterations on the centric side of the on-target Cas9 break that are hallmarks of the formation of chromosome bridges (P9 and S1, after mitosis 3 and 2, respectively, Fig. 2b, c)^6,10^. Cas9-induced chromosome bridges can result from the fusion of unrepaired DSBs on cleaved sister chromatids, which is associated with increased micronucleus formation^6,10^. Indeed, by confocal microscopy we observed chromosome bridges in Cas9-edited embryos (Supplementary Fig. 4a), although the small sample size and limited detection sensitivity in 3D embryos preclude a definitive determination of their frequency.

We determined that chromosome bridges can cause whole chromosome loss. The most parsimonious explanation for the copy number pattern observed in two embryos (blastomeres P9.8, S1.2, and S1.7; Fig. 2b, c) is that on-target Cas9 cleavage resulted in the formation of a dicentric chromosome from the replicated centric portion of the cleaved chromosome. Additionally, after cell division, the replicated acentric fragment was segregated to the same daughter cell as the dicentric chromosome. The co-segregation of these portions of the targeted chromosome results in one daughter containing all copies of one homolog and the other daughter with a monosomy for the other homolog. The exact copy number for the acentric portion of the targeted chromosome depends on whether it segregates to the main nucleus and is replicated, or whether it is segregated into a micronucleus, where it is poorly replicated (Fig. 2b, c). Although alternative paths to the final observed genomic outcomes are possible (Supplementary Fig. 4b, c), these alternatives also require bridge formation as an explanation of the monosomy, which validates the general model. We note that the production of monosomies from dicentric chromosomes is not specific to telocentric chromosomes in the mouse, as a similar model has been invoked to explain some spontaneous monosomies arising in human embryos after in vitro fertilization^19^. Because we analyzed all cells from 8-cell embryos that met our library quality control threshold, our findings support this DNA breakage-induced monosomy model, providing a mechanistic explanation for the recently demonstrated whole targeted chromosome loss resulting from Cas9-derived DSBs^5^.

## Discussion

In summary, we report the most detailed molecular analysis to date of the formation and propagation of complex chromosome alterations after genome editing-mediated and spontaneous missegregation events in the mouse embryo. In contrast with other recent studies^4,5,20^, we are able to deeply sequence most cells from 8-cell embryos, allowing us to reconstruct the karyotypic history of complex events including micronuclei and chromosome bridge formation. Importantly, even the ~2-3-fold increase in micronucleation events after Cas9 treatment can be biologically significant due to the ongoing instability and breakage amplification that occurs after chromosome breakage leads to either micronucleation or further bridge formation^10^. Ongoing instability after an initial break is verified by our identification of multiple missegregation events resulting from micronuclei and chromosome bridges. These types of events have also been recently detected using cell lines^6^. Furthermore, we elucidate a chromosome bridge-mediated mechanism for whole chromosome missegregation that explains the puzzling whole chromosome monosomies observed after genome editing in the embryo^5^. Although we did not detect chromothripsis in the three embryos that our sequencing data indicate contained micronuclei (Supplementary Fig. 5), the small sample size does not exclude chromothripsis as a potential outcome of editing in embryos. Indeed, the chromosome and nuclear aberrations we describe here share features that are well-established to generate chromothripsis in other cell types. Moreover, human patients with complex congenital disease due to chromothripsis have been identified, demonstrating that cells with chromothripsis occurring in the early embryo can undergo clonal expansion during human development^21^.

## Methods

### Mouse handling and CRISPR-Cas9 treatments in embryos

All experiments using mice were carried out with approval from the MIT Committee on Animal Care (CAC) under protocol number 1019-029-22. Experiments were carried out under the supervision of the Division of Comparative Medicine (DCM) at MIT, which provides centralized management of the animal facility at the Whitehead Institute for Biomedical Research. The mouse facility conforms to federal guidelines (Animal Welfare Assurance Number A3125-01), and MIT is accredited by the Assessment and Accreditation of Laboratory Animal Care (AAALAC). Routine bedding, food, and water changes were performed by DCM. Mice were housed in a centrally controlled environment with a 14-h light/10-h dark cycle, temperature of 20–22.2 °C, and humidity of 30–50%.

For the imaging experiments we used B6D2F2 embryos, derived from mating superovulated 8–10-week-old B6D2F1 females to 6–9-month-old B6D2F1 stud males (B6D2F1 is a cross of C57BL/6J females to DBA2 males). For the single-cell whole-genome sequencing analysis, F1 embryos resulting from mating superovulated 4–5-week-old C57BL/6J females to 129/SvJae males were used in order to distinguish the two parental alleles.

Superovulation was induced by an intraperitoneal injection of 7.5 IU PMSG (Pregnant Mares Serum Gonadotropin, Prospec), followed by an injection of 7.5 IU hCG (human Chorionic Gonadotropin, Prospec) and mated to either 129/SvJae or B6D2F1 male stud mice. The morning after mating, copulatory plugs were monitored and pronuclear stage embryos were harvested 20 h post hCG in M2 medium (Cytospring). Immediately after harvesting, embryos were electroporated in a mix containing 100 ng Cas9 Protein (Sigma) and 50 ng/μL synthetic gRNA (Synthego) using a BEX electroporator (Cuy21Edit). Subsequently, embryos were cultured in a 5% CO_2_, 37 °C, humidified incubator, in KSOM-AA (Cytospring) for ~48 h, at which point the embryos had reached the 8-cell stage. For sequences and description of the gRNAs used, see Supplementary Data 2. Note that the synthetic gRNAs are modified with 2′-O-Methyl bases at the first and last three bases and with phosphorothioate bonds between the first three and last two bases.

### Confocal imaging of mouse embryos and micronucleus count

Embryos were collected as described above and washed in PBS -Ca2^+^/Mg2^+^, that was enriched with 0.1% Polyvinylpyrrolidone K90 (PVP MW 360, MP Biosciences). PVP was added in order to prevent adhesion to the pipet walls. Embryos were subsequently fixed in 4% paraformaldehyde (Electron Microscopy Sciences) in PBS-PVP, for 10 min at room temperature. Immediately after fixation, embryos were rinsed twice in PBS-PVP and stored at 4 °C until the next step.

The embryos were transferred within a drop of ~50 μL of Vectashield HardSet Antifade Mounting Medium with DAPI (Vector Laboratories) to a 35 mm gridded μ-Dish (Ibidi) and mounted without a coverslip. The gridded dish was used to ensure that each embryo was imaged only once. Confocal images were collected from distinct embryo samples using Metamorph software (v. 7.10.2.240, Molecular Devices) on a Nikon Ti-E inverted microscope with a Yokogawa CSU-22 spinning disk head with the Borealis modification. Confocal stacks were collected covering the whole z-focal plane of the embryo, at ~0.7–1 µm spacing using a CoolSnap HQ2 CCD camera (Photometrics) and a ×40/1.40 NA LWD objective (Nikon). Embryos with prominent evidence of cellular fragmentation or with multiple blastomeres dividing were not included in the analysis, which comprised fewer than 5% of samples.

### DNA synthesis analysis and image quantification

5-ethynyl-2′-deoxyuridine (EdU) incorporation in newly synthesized DNA was used to study DNA replication in micronuclei of embryos after Cas9/*Scn9a* gRNA treatment. B6D2F2 embryos were transferred into embryo culture medium supplemented with 10 μM EdU at 44 h post hCG on E2.5. EdU was detected using the Click-iT Plus EdU Alex Fluor 488 Imaging Kit (Life Technologies).

All solutions used were supplemented with 0.1% PVP. Incubation in the presence of EdU was allowed to proceed for 5 h, in a CO_2_ incubator, at which point the embryos were fixed for 10 min in 4% paraformaldehyde in PBS. After rinsing three times in PBS containing 3% BSA (PBS-BSA), permeabilization was performed by incubating the embryos in 0.5% Triton X-100 in PBS-BSA. Embryos were rinsed again three times followed by incubation in the Click-iT reaction cocktail for 30 min in the dark and two final rinses with PBS-BSA. The embryos were then mounted in Vectashield HardSet Antifade Mounting Medium with DAPI (Vector Laboratories) on a 35 mm gridded μ-Dish (Ibidi) for subsequent confocal imaging on a Ti2 inverted microscope with a CSU-W1 spinning disk system (Nikon), a Zyla 4.2 sCMOS camera (Andor), and a ×40/1.15 NA LWD objective as described in “Confocal imaging of mouse embryos and micronucleus count”.

For the quantification of DNA replication in the micronuclei we performed image analysis using NIS-Elements AR 5.20.00 (Nikon) and ImageJ 1.52n. Briefly, the single best focal plane was selected for visualization of the micronucleus, and nuclear segmentation was performed based on DAPI staining. If segmentation was unable to detect the primary nucleus because it was on a different imaging plane, a best plane image of the primary nucleus was separately used to quantify DNA replication in the primary nucleus. Segmented nuclei were converted to a mask, and the mask was refined manually if needed, using the “Watershed” or “Draw” functions. Masks were then applied to the best slice images to quantify mean fluorescence intensity of DAPI and EdU. Background was subtracted based on the mean fluorescence intensity of a rectangular region near the nuclei being measured. Finally, EdU values were normalized to the DAPI signal, yielding the density of EdU incorporation. Only samples with ongoing DNA replication in the primary nucleus, operationally defined as a background subtracted primary nucleus EdU intensity ≥10 a.u., were considered.

### Single blastomere dissociation and whole-genome sequencing

To dissociate single cells from 8-cell stage embryos, embryos were washed in PBS-PVP (0.01% Polyvinylpyrrolidone K90, MP Biosciences) and then the zona pellucida was removed following a brief incubation (30 s – 1 min at room temperature) in Acid Tyrode’s solution (Sigma). Embryos were washed in PBS-PVP twice before the dissociation process. Dissociation was accomplished by brief incubation (5 min at 37 °C) in TripleE enzyme (Gibco), followed by washing in PBS-PVP and pipetting repeatedly through a narrow pipet until all cells dissociated. Each individual cell was then transferred in a PCR tube containing 4 μL of PBS and frozen on dry ice.

Whole-genome amplification was performed using the REPLI-g Single Cell kit (Qiagen). Lysis and MDA were performed in PCR tubes containing single blastomeres according to the manufacturer’s protocol except that the amplification was terminated after 80 min. The amplified DNA was then transferred to 96-well plates and stored at 4 °C for a maximum of 24 h. DNA purification was performed in the 96-well plates using an AMPure XP kit (Beckman Coulter) and resuspended in 100 μL TE. Purified DNA was then sheared by sonication (Covaris E220) into ~500 bp fragments. Sheared DNA was then processed by a KAPA LTP Library Preparation Kit (KAPA Biosystems) for multiplexed next-generation sequencing on HiSeq 2500 and NovaSeq 6000 platforms (Illumina).

### gRNA off-target analysis

The CRISPOR program^22^ (http://crispor.tefor.net) was used to predict off-target sites for both gRNAs used in this study. The “Mus musculus (mm10)” mouse reference genome was used according to the guidelines of the on-line tool. Description of the “MIT offtarget” and “CFD offtarget” scores is provided in Concordet et al.^22^. The off-target sites with three or fewer mismatches are shown in Supplementary Fig. 2 for both gRNAs. In addition to CRISPOR, the Cas-OFFinder tool^23^ (http://www.rgenome.net/cas-offinder/) was also used to detect off-targets with three or fewer mismatches for both gRNAs and no additional site was predicted other than the ones identified by CRISPOR (for the predicted off-target sites see Supplementary Fig. 2).

### Bioinformatics pipeline

Bioinformatics analysis for the present manuscript was performed using a modified re-implementation of the methods in Zhang et al.^7^ that is described in the following sections. The GRCm38 p6 build of the mouse reference genome assembly (NCBI) was used for all analysis described below. All copy number plots were generated using ggplot2 version 3.3.3.

### Alignment and preprocessing

Samples were aligned to the mouse reference genome using bwa mem (0.7.17). Library metadata and sample information was added using samtools 1.10 “addreplacerg” sub-command. Read duplicates were then marked using picard “MarkDuplicates” (packaged with GATK v 4.1.0). Finally, reads were sorted in coordinate order using GATK “SortSam”. Alignment summary metrics, insert size metrics, sequencing artifact metrics, and GC bias metrics were obtained using GATK “CollectMultipleMetrics”.

### Read depth-based copy number analysis

Read counts were collected in 10 kb bins using GATK “CollectReadCounts”, and then re-binned into 50 kb bins as the most granular level of copy number analysis. For the low-pass sequencing, we further re-binned into 250 kb bins as we found that 50 kb bins were too noisy to allow robust normalization of locus-specific variability in read depth. Bin-level coverage was then normalized in each sample by dividing by the per-sample median across all bins, and any samples with median coverage 0 across all bins were dropped from further analysis. Additionally, any bins with coverage <25% of the genome-wide median in >80% of samples were excluded from downstream steps. Finally, the bin-level copy number estimates were combined into one of several larger bin sizes (250 kb, 1 Mb, 2 Mb, or 5 Mb) in order to perform refined copy number analysis at multiple levels of resolution. The mean normalized coverage was computed within each of these bins and then divided by the median value across all samples to account for locus-specific coverage biases.

We noticed that even after normalization by the median coverage across all samples, a subset of samples exhibited a characteristic pattern of coverage bias that we found correlated strongly with replication timing. We used the E/L Repli-Seq data for mESC cells^24^ to normalize out this bias using locally estimated scatterplot smoothing (LOESS). Copy number estimates were called on the normalized depth-based copy number data using circular binary segmentation^25^.

Samples with high genome-wide variability in copy number profiles were excluded if the median absolute deviation of copy number across bins was above a manually determined threshold value (0.15 for “HiSeq”, 0.3 for “NovaSeq” samples).

### Genotyping of SNPs specific for parental haplotype

To implement haplotype-aware copy number analysis, we began with a list of SNPs present in the Mouse Genomes Project v5^26^ data for the 129S1/SvImJ line, assuming that this was the most closely related to our 129/SvJae line, compared to the rest of the mouse strains with publicly available genotype data^27^. We reasoned that SNPs present in 129S1/SvImJ and not in the C57BL/6J line enable us to distinguish reads from each of the parental genotypes in our crosses.

We collected allelic read depth counts for each of these 5,132,976 candidate SNP sites using GATK “ASEReadCounter”. Of these, we identified 2,977,810 SNPs that appeared to be present near 50% variant allele fraction (VAF) in our samples (range: 40–60% median VAF). In each case, we inferred that the “reference” allele corresponded to the C57BL/6J genotype and that the “alternate” allele came from the 129/SvJae genotype.

### Haplotype-aware copy number analysis

The allelic depth across SNPs within a 5 kb bin was averaged within each sample to prevent clusters of SNPs with correlated coverage from having excess weight in our haplotype copy number estimates. For a given copy-number bin size (see above), we then computed the sum of allelic depths across all SNPs in a bin to obtain bin-level VAF estimates. The VAF (or 1-VAF) was multiplied by the depth-based copy number to obtain haplotype copy number estimates for the 129/SvJae and C57BL/6J haplotypes, respectively. Additionally, sample ploidy was estimated by taking the mean VAF across all bins in a sample — if min(VAF, 1-VAF)^−1^ was between 2.5 and 3.5, we concluded that the sample was triploid and multiplied the copy number estimates in that sample by 1.5. Otherwise, the sample was assumed to be diploid and no correction was applied. This procedure enabled us to accurately determine haplotype copy number of digynic blastomeres. We also noted the occurrence of rare blastomeres (usually with poor library quality) whose ploidy did not match that of the other blastomeres in the embryo — these were also excluded for purposes of visualization and inference of chromosome segregation events.

After this correction, we observed that all copy number alterations identified from read depth information could be attributed to integer copy number gains or losses of one of the two parental haplotypes (except for three samples presenting with a ½ copy gain or loss of a single chromosome, which can be explained by underreplication of those chromosomes in micronuclei), supporting the overall accuracy of our haplotype phase inference and ploidy estimation.

### Structural variant calling and joint genotyping

We used SvABA 1.1.0 to identify SVs independently in each sample, excluding SV calls from the Mouse Genomes Project data for 129S1/SvImJ, and those supported by fewer than 3 sequencing reads. We also required that at least one supporting read pair had a mapping quality >30. We then used an in-house script to perform joint genotyping of candidate de novo SVs with inter-breakpoint distance >1 Mb across all samples, by tallying split and discordant reads in all samples. Any SVs observed only within one embryo (but possibly in more than one blastomere) were considered for further analysis. This distance filter and joint genotyping step were necessary to exclude a large background of artifactual SV calls. We confirmed that this workflow was able to recapitulate the results of Leibowitz et al.^6^, re-identifying the subset of samples previously reported to have undergone chromothripsis.

### Reporting summary

Further information on research design is available in the Nature Research Reporting Summary linked to this article.

## Supplementary information

Supplementary Information

Description of Additional Supplementary Files

Supplementary Data 1

Supplementary Data 2

Reporting Summary

## Data Availability

The sequencing read data generated in this study have been deposited in the Sequence Read Archive under BioProject PRJNA761020. The raw image data contributing to Figs. 1 and 3 and Supplementary Fig. 4 were not published due to constraints of file size but are available from the corresponding authors upon reasonable request. The micronucleus count and EdU incorporation data generated for Figs. 1b and 3b in this study are provided in the Source Data files. SV and CN breakpoints calls are also available in the Source Data. Source data are provided with this paper.

## Source data

Source Data

## Code availability

Scripts used for sequencing data analysis are available at https://github.com/logan-blaine/scDNA-pipeline.
