# Supplementary material for: Whole chromosome loss and genomic instability in mouse embryos after CRISPR-Cas9 genome editing
Source: Nat Commun. 2021 Oct 6;12:5855. doi: 10.1038/s41467-021-26097-y (PMC8494802; doi:10.1038/s41467-021-26097-y)
Supplement: Supplementary file 1 — Supplementary Information [file 41467_2021_26097_MOESM1_ESM.pdf]

Supplementary Figure 1

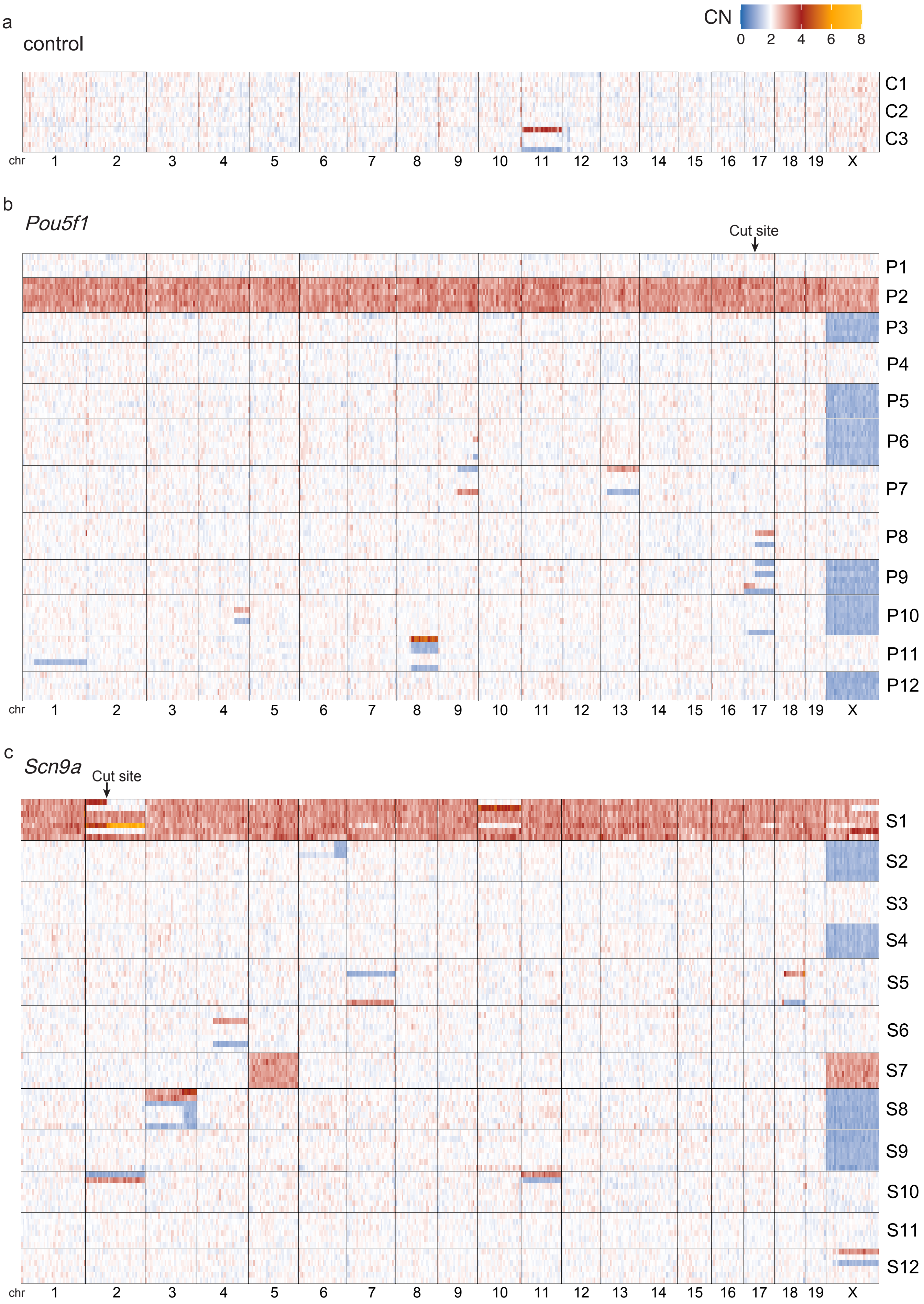

Supplementary Figure 1. Copy number profiles of the blastomeres from all 8-cell stage embryos

Shown are CN heatmaps as in Fig. 1d for the indicated samples. Bin size: 5 Mb. Both male and female embryos were included in the analysis, as indicated by the chromosome X copy number profiles. The Y-chromosome is not shown because of limited mappable sequence. The S1 and P2 embryos are triploid, presumably due to whole genome duplication during oogenesis. For the sequencing metrics and description of the chromosomal events of each embryo see Supplementary Data 1. The blastomere samples are arranged as presented in the Supplementary Data 1, excluding those that do not pass the sequencing quality filters as described in Methods (for a full list of samples see Supplementary Data 1).

We detected segmental CN alterations of greater than 10 Mb size as well as whole-chromosome aneuploidies in 12.5% of the blastomeres in the untreated embryo group, 15.8% in the Pou5f1 and 25.6% in the Scn9a group (see also Supplementary Data 1).

a

Supplementary Figure 2

Embryo	Blastomere	Chromosome	Genomic location (Mb)
P6	P6.4, P6.7	9	108.5
P7	P7.1, P7.5	9	59.5
P7	P7.1	13	20.5
P7	P7.5	13	19.5
**P8**	**P8.4**	**17**	**35.5**
**P8**	**P8.6**	**17**	**34.5**
**P9**	**P9.2, P9.5**	**17**	**34.5**
**P9**	**P9.7**	**17**	**35.5**
P10	P10.3	4	109.5
P10	P10.5	4	110.5
P10	P10.7	17	12.5
P11	P11.5	1	34.5
P11	P11.1, P11.2, P11.3, P11.6	8	43.5
**S1**	**S1.1**	**2**	**65.5**
S1	S1.2	X	81.5
**S1**	**S1.6**	**2**	**66.5**
S1	S1.7	X	80.5
S2	S2.1, S2.2	6	110.5
S2	S2.3	6	109.5
S5	S5.3	18	32.5
S5	S5.8	18	29.5
S8	S8.1, S8.4, S8.5, S8.6	3	116.5

b

Pou5f1 gRNA sequence

ACCCACCAAAGAGAACGCCCAGG

Offtarget Sequence	Mismatch Position	Mismatch Count	MIT Offtarget Score	CFD Offtarget Score	chromosome	start	end	strand	Notes
ACCAACCAAAGAGAAAGCTCAGG	...*...........*..*.	3	0.17071642	0.38866397	chr5	62822971	62822993	-	CRISPOR/CasOFF-finder
ACCAACCAAAGAGAAAGCACAGG	...*...........*..*.	3	0.17071642	0.36090226	chr5	63467362	63467384	+	CRISPOR/CasOFF-finder
ACCAACCAAAGAGAAAGCACAGG	...*...........*..*.	3	0.17071642	0.36090226	chrX	61708632	61708654	-	CRISPOR/CasOFF-finder
ACCAACCAAAGAGAAAGCACAGG	...*...........*..*.	3	0.17071642	0.36090226	chr1	8605761	8605783	+	CRISPOR/CasOFF-finder
ACCCAGCAGAGAGAAAGCCCAGG	.....*..*......*....	3	0.17896778	0.33333333	chr8	117951595	117951617	-	CRISPOR/CasOFF-finder
ACCCACCAAAGAGTACACACAGG	.............*..*.*.	3	0.04384796	0.21333333	chr11	13834429	13834451	+	CRISPOR/CasOFF-finder
ACCAACCAAAGAGCAAGCCCAGG	...*.........*.*....	3	0.07620219	0.18045113	chr9	89145746	89145768	+	CRISPOR/CasOFF-finder
ACCCACCAAAGAGAAACCCATGG	...............**..*	3	0.06700652	0.11764706	chr4	4680084	4680106	-	CRISPOR/CasOFF-finder
ACCCAGCAAAGAGAAACCCCAGG	.....*.........**...	3	0.11277021	0.11764706	chrX	41813280	41813302	-	CRISPOR/CasOFF-finder
CCCCACCACAGAGAACCCCCTGG	*.......*.......*...	3	0.78826543	0.1152461	chr5	114028036	114028058	+	CRISPOR/CasOFF-finder
AGCCACCAATGAGAAGGCCCCGG	.*.......*.....*....	3	0.49914229	0.09443242	chr15	88819494	88819516	-	CRISPOR/CasOFF-finder
AACCACCAAAGAGAACGCATGGG	.*................**	3	0.47000847	0.09350649	chr9	90389717	90389739	-	CRISPOR/CasOFF-finder
ACCCACCCAAGAGAACGTCTTGG	.......*.........*.*	3	0.2430216	0.08265306	chr6	39017142	39017164	+	CRISPOR/CasOFF-finder
ACCAACCAAAGAGCACGCACAGG	...*.........*....*.	3	0.14788806	0.0773362	chrX	150434475	150434497	+	CRISPOR/CasOFF-finder
ACCAACCAAAGAGCACGCACAGG	...*.........*....*.	3	0.14788806	0.0773362	chr6	85167129	85167151	+	CRISPOR/CasOFF-finder
ACCCACCAAAGAGCATACCCAGG	.............*.**...	3	0.02288997	0.06153846	chr10	121060566	121060588	-	CRISPOR/CasOFF-finder
ACCAACCAAAGAGCATGCCCAGG	...*.........*.*....	3	0.07620219	0.05552342	chr12	13905999	13906021	-	CRISPOR/CasOFF-finder
ACCAACCAAAGAGCATGCCCAGG	...*.........*.*....	3	0.07620219	0.05552342	chr10	102683748	102683770	+	CRISPOR/CasOFF-finder
ACCAGCCAAAGAGAACTCCCCAG	...**...........*...	3	0.22895149	0.03929825	chr6	99974344	99974366	+	CRISPOR
AGACAGCAAAGAGAACGCCCAGA	.**..*..............	3	0.2895037	0.02093398	chr18	37748618	37748640	+	CRISPOR

Scn9a gRNA sequence

ATGTGGATGTCAGTTTATAGAGG

Offtarget Sequence	Mismatch Position	Mismatch Count	MIT Offtarget Score	CFD Offtarget Score	chromosome	start	end	strand	Notes
ATGTGGTTGTCAGTTTATAGAAG	......*.............	1	13.66	0.113425926	chr17	45890918	45890940	+	CRISPOR
AGGTAAATGTCAGTTTATAGAGG	.*..**..............	3	1.46807152	0.728	chr19	25457169	25457191	-	CRISPOR/CasOFF-finder
ACCTGGATGTCAATTTATAGTGG	.**........*........	3	1.074082667	0.304311074	chr1	96239792	96239814	+	CRISPOR/CasOFF-finder
ATGTAGAGGTCATTTTATAGAGG	....*..*....*.......	3	1.034177215	0.190666667	chr1	125222180	125222202	+	CRISPOR/CasOFF-finder
ATGTGTATGTCTGTTTATATGGG	.....*.....*.......*	3	0.391104507	0.155555555	chr1	86809333	86809355	-	CRISPOR/CasOFF-finder
ATATGTAAGTCAGTTTATAGGAG	..*..*.*............	3	0.289503704	0.103703704	chr19	4539524	4539546	+	CRISPOR
ATGTGCATGTGTGTTTATAGTGG	.....*....**........	3	0.420190723	0.056818182	chr5	25161870	25161892	+	CRISPOR/CasOFF-finder
ATGTGTATGTGTGTTTATAGAGG	.....*....**........	3	0.420190723	0.055555556	chr1	126396727	126396749	-	CRISPOR/CasOFF-finder
ATGTGGCTGTCAGTTTCTATTGG	......*.........*..*	3	0.326039509	0.054044118	chr5	133517284	133517306	-	CRISPOR/CasOFF-finder
ATGTGGAAGTCAGTTTTTGGAGG	.......*........*.*.	3	0.341366667	0.04	chr9	45030427	45030449	+	CRISPOR/CasOFF-finder
AACTGGATGTCAGTTTGTAGAAG	.**.............*...	3	0.239223549	0.014889585	chr3	11557800	11557822	-	CRISPOR
ATGTAGATGTGAGTGTATAGTGG	....*.....*...*.....	3	0.418675556	0.010833333	chr10	64366158	64366180	+	CRISPOR/CasOFF-finder
ATATTGTTGTCAGTTTATAGGGA	..*.*.*.............	3	0.326828148	0.006835937	chr1	101430663	101430685	-	CRISPOR

Supplementary Figure 2. Compendium of copy number breakpoints identified in the study and comparison to predicted off-target Cas9 cleavage

(a) Genomic locations of the sites where CN transitions were detected, indicating DNA breakpoints. Shown are the data for all the embryos sequenced to high depth ("NovaSeq", see Supplementary Data 1). The blastomeres from which the breakpoints were detected are listed in the "samples" column. Note: the breakpoint sites in the sequenced embryos are approximate genomic locations identified by circular binary segmentation. We considered sites within 1 Mb as the same breakpoint event. None of the 1 Mb windows for chromosomal breakpoints were predicted to contain off-target sites for the gRNAs. Bold text indicates breakpoints associated with the on-target cut site of the respective gRNA. Source data are provided as a Source Data file.

(b) List of off-target sites for *Pou5f1* and *Scn9a* gRNAs using CRISPOR and CasOff-finder. Only the off-target sites with less than three mismatches are shown. Cutting frequency determination ("CFD") and the MIT specificity ("MIT", from the CRISPR Design website; http://crispr.mit.edu) scores are shown, as described in Ref. 22.

Supplementary Figure 3

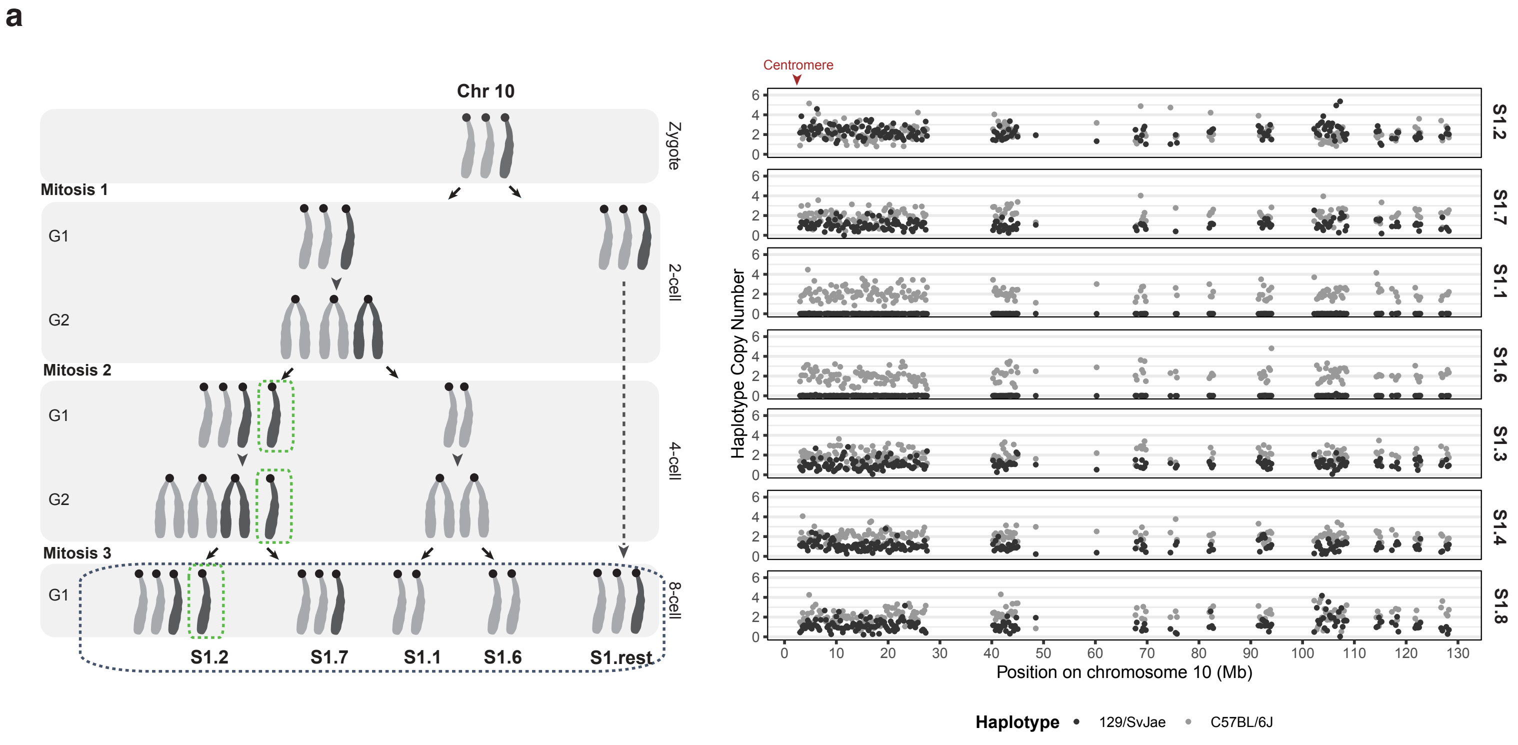

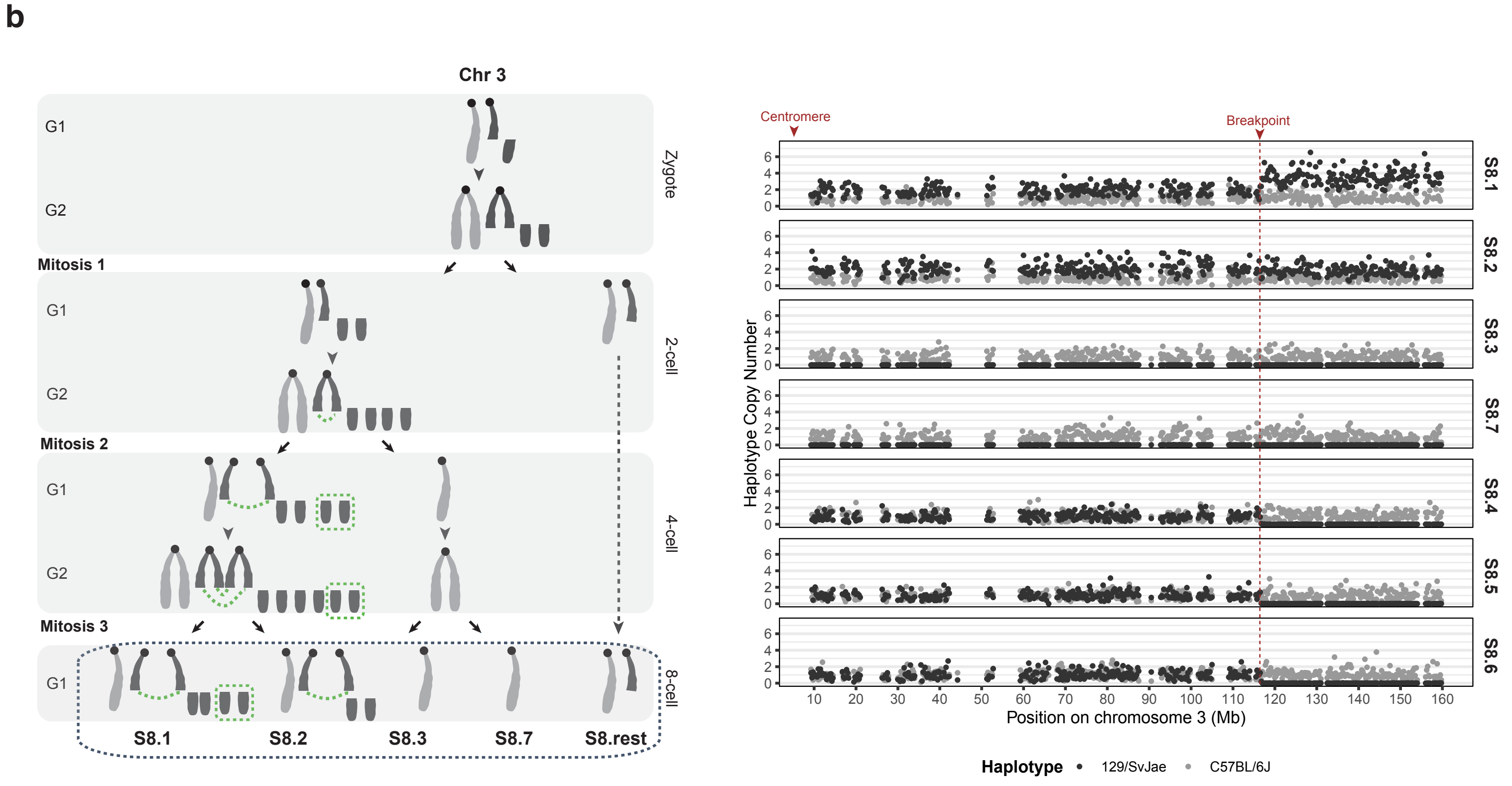

Supplementary Figure 3. Spontaneous micronucleus formation in embryos, unrelated to on-target Cas9 cleavage

(a) Left, scheme to explain the copy number (CN) pattern for blastomeres of the S1 embryo (*Scn9a* gRNA). In this example, a whole-chromosome containing micronucleus formed involving a different chromosome than the targeted chromosome. Right: haplotype CN scatter plots (see also Fig. 2c) for blastomeres from this embryo (250 kb bins) for the targeted chromosome. Labelling and color schemes as in Fig. 2.

(b) Left, scheme to explain the CN pattern for blastomeres of the S8 embryo (*Scn9a* gRNA). A break in chromosome 3 occurred at the zygote's G1 stage (or alternatively two independent cuts at the same locus of both sister chromatids at the G2) resulting in missegregation of both telomeric acentric fragments to the primary nucleus of one daughter cell. Based on the odd numbered DNA CN indicating underreplication, we infer that the micronucleus formed after Mitosis 2. In this scenario, a chromosome bridge explains the co-segregation of the centromeric segments to the same daughter. An alternative scenario explaining these CN profiles would require breakage of only one of the two sister chromatids before Mitosis 1 and a subsequent cut of one chromatid of the same haplotype at the next interphase. Right, CN plots as in (a). Labelling and color schemes as in Fig. 2.

Supplementary Figure 4

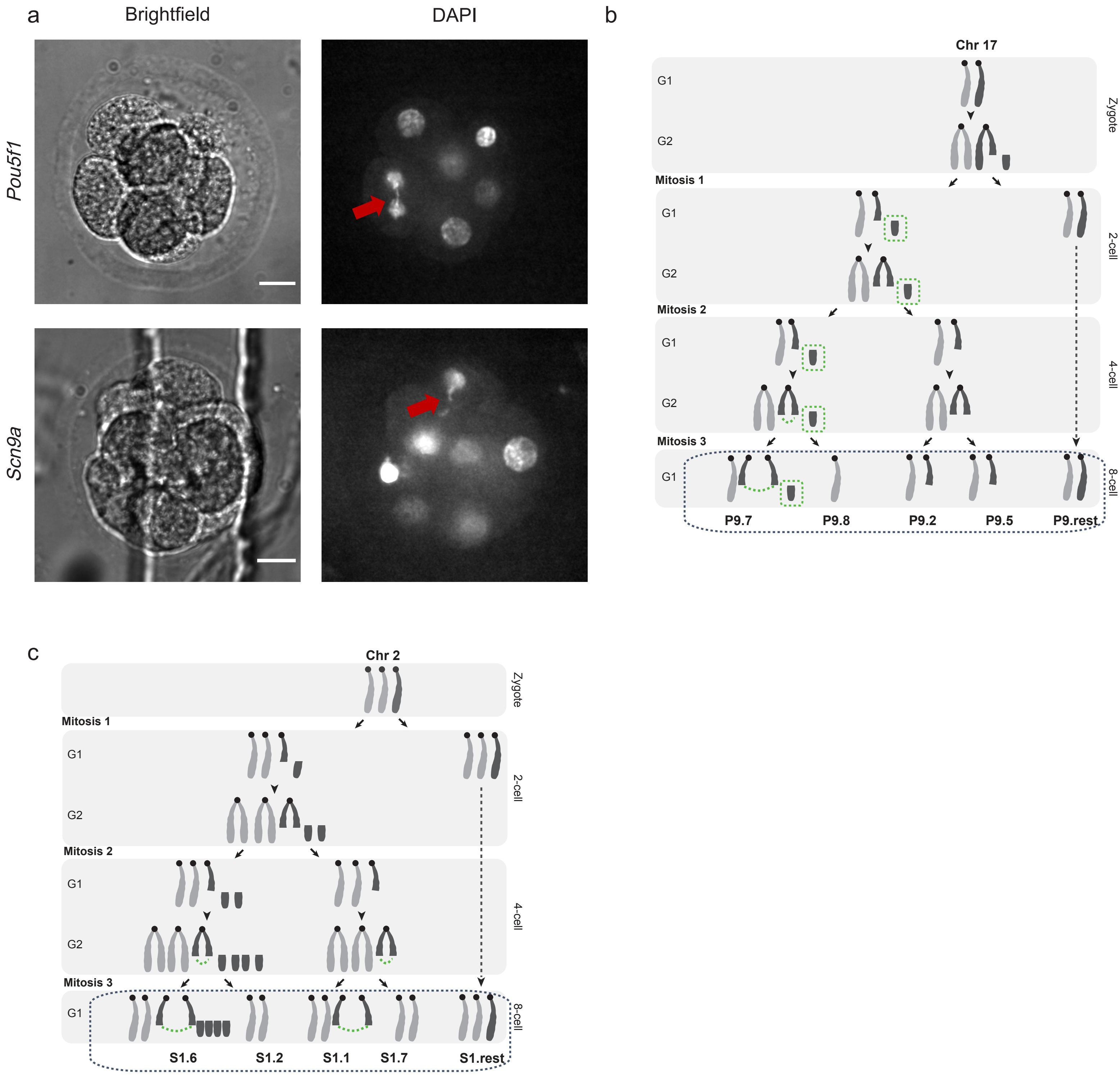

Supplementary Figure 4. Alternative explanation for CN patterns in P9 and S1 embryos and example chromosome bridges in 8-cell embryos

(a) Cytological evidence of chromosome bridge formation in mouse embryos upon CRISPR-Cas9 treatment. Shown are maximum intensity projections from five z-focal plane confocal images of 8-cell stage mouse embryos after Cas9 treatment (top: *Pou5f1* gRNA; bottom: *Scn9a* gRNA. Red arrows: chromosome bridge. Scale bar: 15 μm. We note that the limited detection sensitivity in 3D embryos preclude a definitive determination of their frequency, and these images were collected over a single experiment.

(b) Alternative explanation for CN patterns seen in Fig. 2. In this scenario for the P9 embryo, one sister chromatid of one homologue is cut in a G2 zygote. The illustrated pattern of segregation would explain the observed CN pattern in this embryo (Fig. 2b), but unlike the scenario in Fig. 2b, the 8-cell blastomere would have been harvested in the G1 phase. Although we cannot exclude this model, we disfavor it because if Cas9 cuts in a G2 cell it is common for it to cleave both sisters[6]. Labelling and color schemes as in Fig. 2.

(c) Alternative explanation for CN patterns seen in Fig. 2. In this scenario for the S1 embryo, instead of the chromosome bridge forming before Mitosis 2, both cells independently form bridges before Mitosis 3.

In all examples derived from on-target cleavage, the chromosomal missegregation events occurred as early as in the second (P9 and S1) or third mitosis (P8), suggesting that the CRISPR-Cas9 cut was introduced in the 2-cell cleavage stage, consistent with other studies[2]. It is likely that one homologue was cleaved in G1, and after DNA replication two acentric sister chromatid fragments segregated to one daughter.

Alternatively, but less likely, two independent cuts on each sister chromatid may have occurred in a G2 phase cell.

Supplementary Figure 5

Pou5f1

Blastomere	Embryo	Chromosome	# of SVs
P6.5	P6	chr16	1
P7.1	P7	chr11	1
P8.1	P8	chr4	1
P8.1	P8	chr16	4
P8.2	P8	chr4	1
P8.4	P8	chr17	1
P8.4	P8	chr18	1
P8.7	P8	chr13	1
P9.2	P9	chr2	1
P9.2	P9	chr3	1
P9.4	P9	chr13	1
P9.4	P9	chr7	1
P9.8	P9	chr6	1
P10.1	P10	chr6	1
P11.1	P11	chr8	1
P11.2	P11	chr14	1
P11.5	P11	chr10	1

Scn9a

Blastomere	Embryo	Chromosome	# of SVs
S1.2	S1	chr9	1
S1.2	S1	chr12	1
S1.2	S1	chr16	1
S1.2	S1	chr19	1
S1.3	S1	chr2	1
S1.6	S1	chr3	1
S1.6	S1	chr18	1
S1.7	S1	chr9	1
S2.4	S2	chr9	1
S2.5	S2	chr8	1
S2.6	S2	chr10	1
S5.3	S5	chr9	1
S5.6	S5	chr4	1
S5.7	S5	chr5	1
S5.7	S5	chr7	1
S5.7	S5	chr8	1
S7.1	S7	chr7	1
S7.1	S7	chr12	1
S7.1	S7	chrX	1
S7.3	S7	chr17	2
S7.6	S7	chrX	1
S8.7	S8	chr1	1
S8.7	S8	chr9	1
S10.1	S10	chr14	1
S10.5	S10	chr1	1
S10.6	S10	chr1	1
S10.6	S10	chr7	1
S10.6	S10	chr8	1
S10.7	S10	chr3	1
S10.7	S10	chr9	1

Supplementary Figure 5. Absence of clustered rearrangements characteristic of chromothripsis in the analyzed embryos

Lists of the number of structural variants (SVs) detected in the indicated blastomeres based on our SV analysis pipeline for single-cell whole genome sequencing data (the blastomeres that were sequenced to high depth are shown, “NovaSeq”, see Supplementary Data 1). Numbers are shown for chromosomes with at least one SV (n = 1800 total chromosomes from 90 samples passing library quality control checks). We did not detect chromothripsis in any of the blastomeres sequenced. Source data are provided as a Source Data file.

Supplementary Data 1. Copy number analysis of sequenced embryo cells and description of chromosomal events

List of all the blastomeres sequenced per embryo and sequencing quality assessment for both sequencing platforms used (HiSeq and NovaSeq). The filters applied to assess sequencing quality are described in Methods. A description of inferred chromosomal events for each embryo is presented in column “chromosomal events”.

Note: Presence of micronucleus is inferred based on the DNA replication status of the missegregated chromosomal segments. Cases showing defective DNA replication are considered as micronucleation events, however we cannot exclude the possibility that micronuclei with normal DNA replication were present in other cases of chromosome missegregation. NA: not applicable.

Supplementary Data 2. gRNA sequences

List of the sequences and genomic coordinates of the gRNAs used in the study.
