# Supplementary material for: Whole chromosome loss and genomic instability in mouse embryos after CRISPR-Cas9 genome editing
Source: Nat Commun. 2021 Oct 6;12:5855. doi: 10.1038/s41467-021-26097-y (PMC8494802; doi:10.1038/s41467-021-26097-y)
Supplement: Supplementary file 2 — Description of Additional Supplementary Files [file 41467_2021_26097_MOESM2_ESM.pdf]

Title: Supplementary Data 1. Copy number analysis of sequenced embryo cells and description of chromosomal events

Description: List of all the blastomeres sequenced per embryo and sequencing quality assessment for both sequencing platforms used (HiSeq and NovaSeq). The filters applied to assess sequencing quality are described in Methods. A description of inferred chromosomal events for each embryo is presented in column "chromosomal events".

Note: Presence of micronucleus is inferred based on the DNA replication status of the missegregated chromosomal segments. Cases showing defective DNA replication are considered as micronucleation events, however we cannot exclude the possibility that micronuclei with normal DNA replication were present in other cases of chromosome missegregation. NA: not applicable.

Title: Supplementary Data 2. gRNA sequences

Description: List of the sequences and genomic coordinates of the gRNAs used in the study.
