# Supplementary material for: Whole chromosome loss and genomic instability in mouse embryos after CRISPR-Cas9 genome editing
Source: Nat Commun. 2021 Oct 6;12:5855. doi: 10.1038/s41467-021-26097-y (PMC8494802; doi:10.1038/s41467-021-26097-y)
Supplement: Supplementary file 5 — Reporting Summary [file 41467_2021_26097_MOESM5_ESM.pdf]

nature research

Corresponding author(s): David Pellman, Rudolf Jaenisch

Last updated by author(s): Sep 5, 2021

Reporting Summary

Nature Research wishes to improve the reproducibility of the work that we publish. This form provides structure for consistency and transparency in reporting. For further information on Nature Research policies, see our Editorial Policies and the Editorial Policy Checklist.

Statistics

For all statistical analyses, confirm that the following items are present in the figure legend, table legend, main text, or Methods section.

n/a	Confirmed	
☐	☒	The exact sample size (*n*) for each experimental group/condition, given as a discrete number and unit of measurement
☐	☒	A statement on whether measurements were taken from distinct samples or whether the same sample was measured repeatedly
☐	☒	The statistical test(s) used AND whether they are one- or two-sided<br>*Only common tests should be described solely by name; describe more complex techniques in the Methods section.*
☒	☐	A description of all covariates tested
☒	☐	A description of any assumptions or corrections, such as tests of normality and adjustment for multiple comparisons
☐	☒	A full description of the statistical parameters including central tendency (e.g. means) or other basic estimates (e.g. regression coefficient) AND variation (e.g. standard deviation) or associated estimates of uncertainty (e.g. confidence intervals)
☐	☒	For null hypothesis testing, the test statistic (e.g. *F*, *t*, *r*) with confidence intervals, effect sizes, degrees of freedom and *P* value noted<br>*Give P values as exact values whenever suitable.*
☒	☐	For Bayesian analysis, information on the choice of priors and Markov chain Monte Carlo settings
☒	☐	For hierarchical and complex designs, identification of the appropriate level for tests and full reporting of outcomes
☒	☐	Estimates of effect sizes (e.g. Cohen's *d*, Pearson's *r*), indicating how they were calculated

Our web collection on statistics for biologists contains articles on many of the points above.

Software and code

Policy information about availability of computer code

Data collection	Collection of fixed imaging data was performed using Metamorph 7.10.2.240 (Molecular Devices) and NIS-Elements AR 5.20.00 (Nikon). (Nikon).
Data analysis	Statistical analysis and graphical plots were performed using Graphpad Prism 7.0d (Graphpad Software Inc). A custom ImageJ Macro (v 1.51 and 1.52n) was used for quantification of fixed imaging data for the EdU experiment (Fig3). Sequencing data were analyzed using R 4.0.2 custom scripts and genomics figures were generated using the ggplot2 package (version 3.3.3). BWA mem was used for alignment (v 0.7.17). GATK v 4.1.0 was used in read processing SvABA 1.1.0 was used for SV calling.

For manuscripts utilizing custom algorithms or software that are central to the research but not yet described in published literature, software must be made available to editors and reviewers. We strongly encourage code deposition in a community repository (e.g. GitHub). See the Nature Research guidelines for submitting code & software for further information.

Data

Policy information about availability of data

All manuscripts must include a data availability statement. This statement should provide the following information, where applicable:

- Accession codes, unique identifiers, or web links for publicly available datasets
- A list of figures that have associated raw data
- A description of any restrictions on data availability

The Sequencing read data generated in this study have been deposited in the Sequence Read Archive under BioProject PRJNA761020 [http://www.ncbi.nlm.nih.gov/bioproject/761020]. The raw image data contributing to Figure 1, 3 and Supplementary Figure 4 were not published due to constraints of file size but are available from the corresponding authors upon reasonable request. The micronucleus count and EdU incorporation data generated for Figures 1b and 3b in this study are provided in the Source Data files. SV and CN breakpoints calls are also available in the Source Data.

Field-specific reporting

Please select the one below that is the best fit for your research. If you are not sure, read the appropriate sections before making your selection.

☒ Life sciences ☐ Behavioural & social sciences ☐ Ecological, evolutionary & environmental sciences

For a reference copy of the document with all sections, see nature.com/documents/nr-reporting-summary-flat.pdf

Life sciences study design

All studies must disclose on these points even when the disclosure is negative.

Sample size	The sample sizes for each experiment were not predetermined using statistical analyses. Sample sizes were chosen based on sample sizes used in other studies of mouse embryos using imaging or sequencing (Vázquez-Diez et al., PNAS 2016) and based on the feasibility of the experiments.
Data exclusions	Embryos with prominent evidence of cellular fragmentation or with multiple blastomeres dividing were not included in the imaging analyses, which comprised fewer than 5% of samples. This criteria was set before analysis. In the sequencing analysis, samples with high genome-wide variability in copy number profiles were excluded using manually determined cutoffs. Samples were excluded from copy number visualization if either of the following two conditions were met: 1. The median absolute deviation of bin-level copy number estimates was greater than a given threshold (0.15 for HiSeq data, 0.3 for NovaSeq data) 2. The estimated ploidy of the sample did not match the rest of the cells in the embryo. The cutoffs for these exclusions were decided upon during analysis, as the cutoffs were dependent upon data quality for minimizing sequencing sample noise.
Replication	The experimental findings of micronucleus formation were reproducible through 5-6 experiments per treatment condition (and 2 experiments for untreated embryos). Sequencing studies were consistent with previous reports (Vázquez-Diez et al., 2016; Leibowitz et al., 2021), and chromosome loss, missegregation, and underreplication were all observed in more than one sample. EdU experiment results are consistent with previous publications in different cell lines (Zhang et al., 2015, Leibowitz et al., 2021. Hatch et al., 2013).
Randomization	Embryos were divided randomly into each treatment condition.
Blinding	The imaging analysis of micronucleus formation in embryos was performed by blinded user to the groups analyzed. The sequencing analysis of structural variants was performed without prior knowledge of the experimental group for each cell.

Reporting for specific materials, systems and methods

We require information from authors about some types of materials, experimental systems and methods used in many studies. Here, indicate whether each material, system or method listed is relevant to your study. If you are not sure if a list item applies to your research, read the appropriate section before selecting a response.

Materials & experimental systems

n/a	Involved in the study
☒	☐ Antibodies
☒	☐ Eukaryotic cell lines
☒	☐ Palaeontology and archaeology
☐	☒ Animals and other organisms
☒	☐ Human research participants
☒	☐ Clinical data
☒	☐ Dual use research of concern

Methods

n/a	Involved in the study
☒	☐ ChIP-seq
☒	☐ Flow cytometry
☒	☐ MRI-based neuroimaging

Animals and other organisms

Policy information about studies involving animals; ARRIVE guidelines recommended for reporting animal research

Laboratory animals	Mice, C57Bl/6J, B6D2F1 and F2, DBA/2, 129Sv/Jae, Male, Female, 8 weeks to 9 months of age
Wild animals	No wild animals were used in this study.
Field-collected samples	No field collected samples were used in this study.
Ethics oversight	All experiments using mice were carried out with approval from the MIT Committee on Animal Care (CAC) under protocol number 1019-029-22. Experiments were carried out under the supervision of the Division of Comparative Medicine (DCM) at MIT, which provides centralized management of the animal facility at the Whitehead Institute for Biomedical Research. The mouse facility conforms to federal guidelines (Animal Welfare Assurance Number A3125-01), and MIT is accredited by the Assessment and Accreditation of Laboratory Animal Care (AAALAC).

Note that full information on the approval of the study protocol must also be provided in the manuscript.
